# Supplementary material for: HC-HA/PTX3 from Human Amniotic Membrane Induced Differential Gene Expressions in DRG Neurons: Insights into the Modulation of Pain
Source: Cells. 2024 Nov 15;13(22):1887. doi: 10.3390/cells13221887 (PMC11592720; doi:10.3390/cells13221887)
Supplement: Supplementary file 1 [file cells-13-01887-s001.zip › Supplemental Figures.pdf]

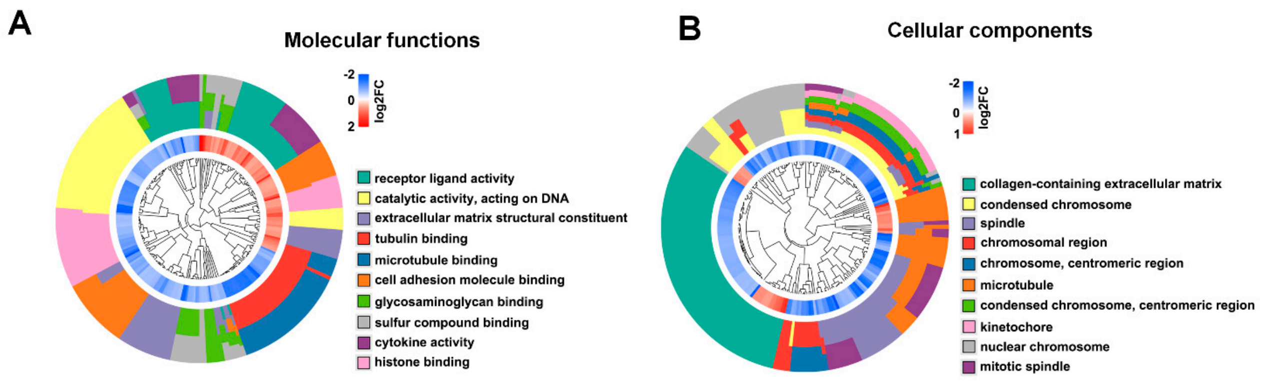

Supplemental Figure S1. HC-HA/PTX3 (HHP) treatment-induced differentially expressed genes are enriched for neuroactive ligand-receptor interaction and extracellular matrix (ECM).

(A-B) The cluster plots are visualized by R package GO plot for the top 10 GO annotations. Differentially expressed genes (DEGs) in cultured wild-type (WT) mouse DRG neurons after 24 hours of HC-HA/PTX3 treatment (15 μg/mL) are enriched for neuroactive ligand-receptor interaction and extracellular matrix (ECM). The plots show a circular dendrogram of the clustering of all the genes included in the top 10 GO terms. The inner ring indicates the color-coded $\log_2$FC. The outer ring represents the assigned molecular function (A) and cellular component terms (B).

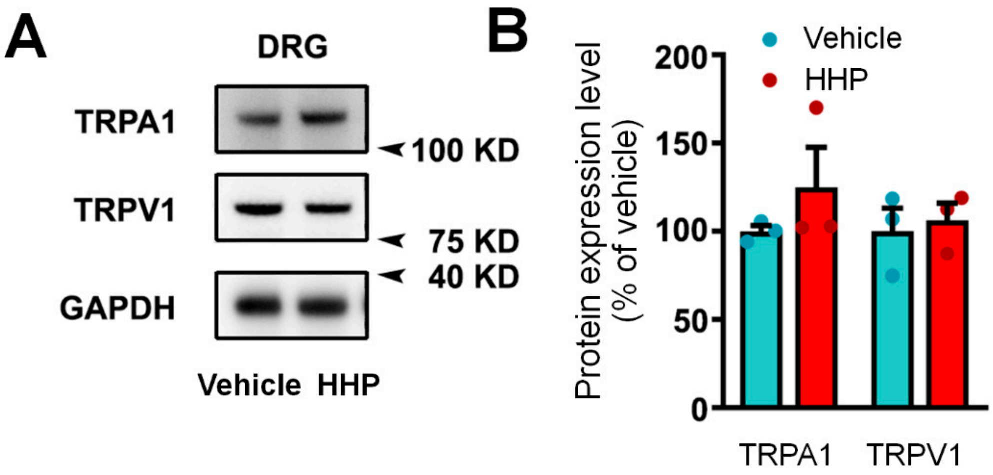

Supplemental Figure S2. Long-term HC-HA/PTX3 treatment did not affect the expression of TRPA1 and TRPV1 in the DRG. (A) Western immunoblotting images (cropped blots, full-length blots are presented in Supplementary File 1) show the expression of TRPA1 and TRPV1 in cultured wild-type (WT) mouse DRG neurons after 24 hours of HC-HA/PTX3 treatment (15 µg/mL). **(B)** The quantification of protein levels, which were normalized to GAPDH (37 kDa). The mean TRPA1 and TPRV1 protein levels in the vehicle group were considered to be 100%. N=3 /group. Data are mean ± SEM. Unpaired Student's t-test. P>0.05.
