# Supplementary material for: HC-HA/PTX3 from Human Amniotic Membrane Induced Differential Gene Expressions in DRG Neurons: Insights into the Modulation of Pain
Source: Cells. 2024 Nov 15;13(22):1887. doi: 10.3390/cells13221887 (PMC11592720; doi:10.3390/cells13221887)

Full unedited gel for Figure 3D

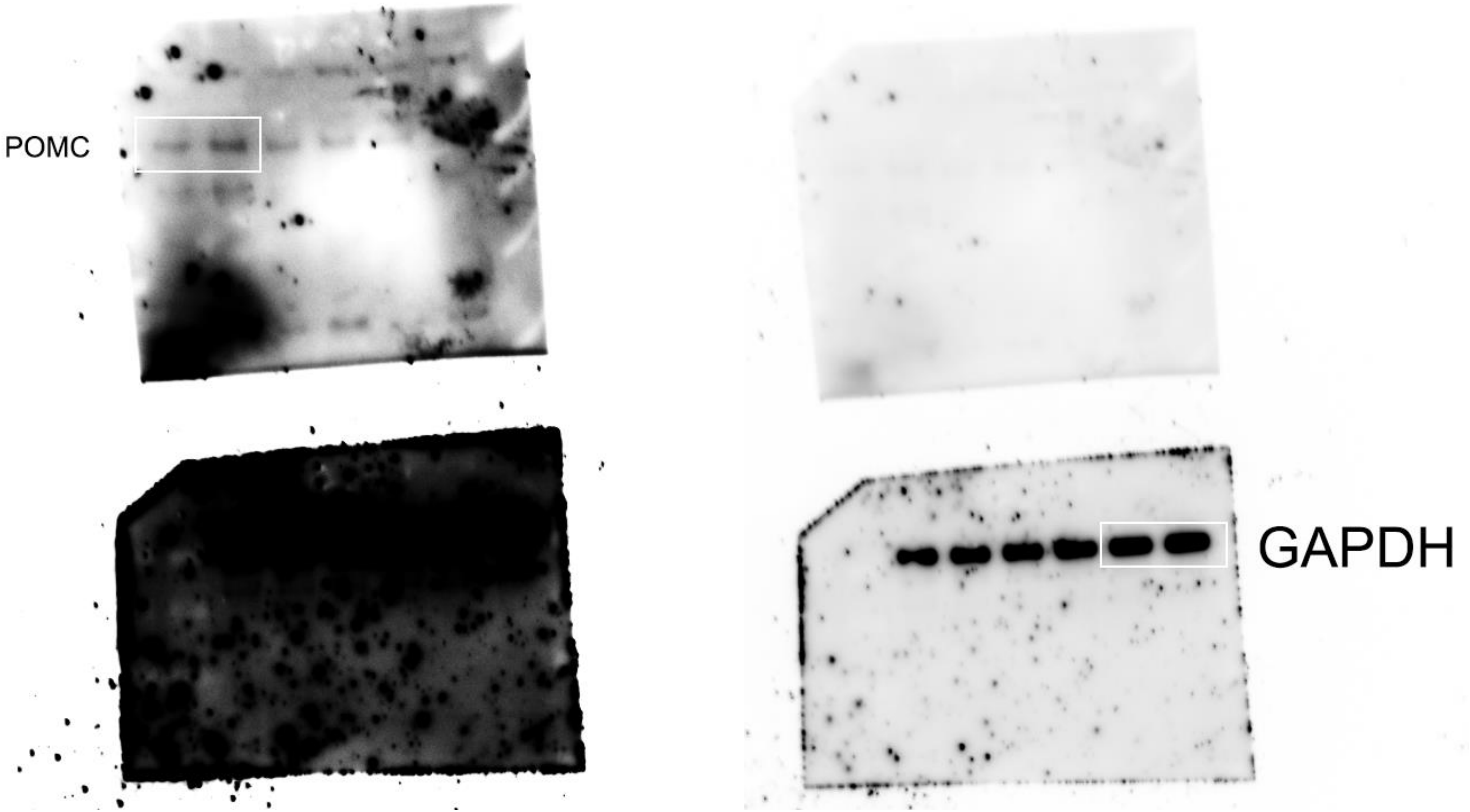

Full unedited gel for Supplemental Figure 2A- TRPA1

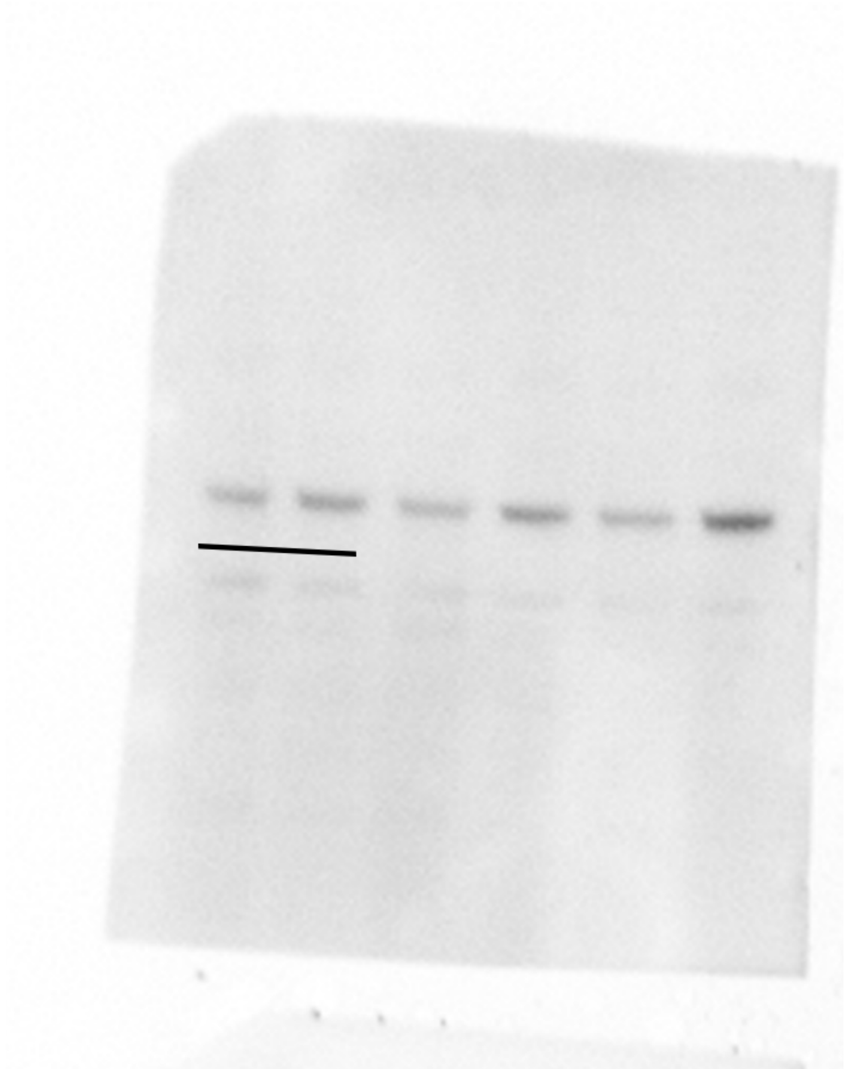

Full unedited gel for Supplemental Figure 2A- TRPV1

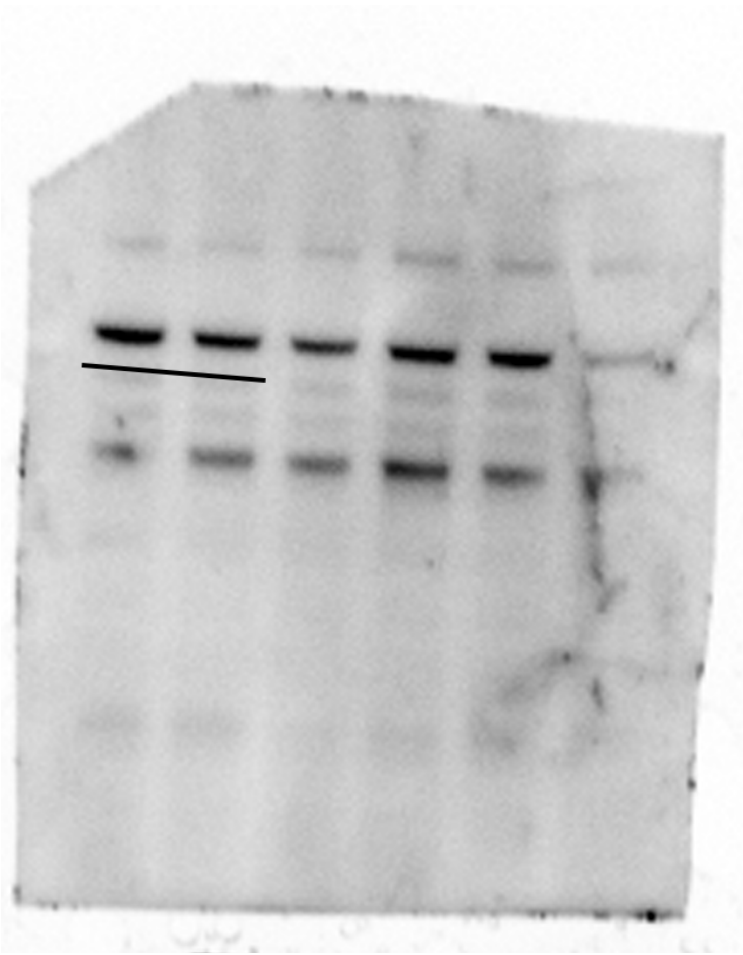

Full unedited gel for Supplemental Figure 2A- GAPDH

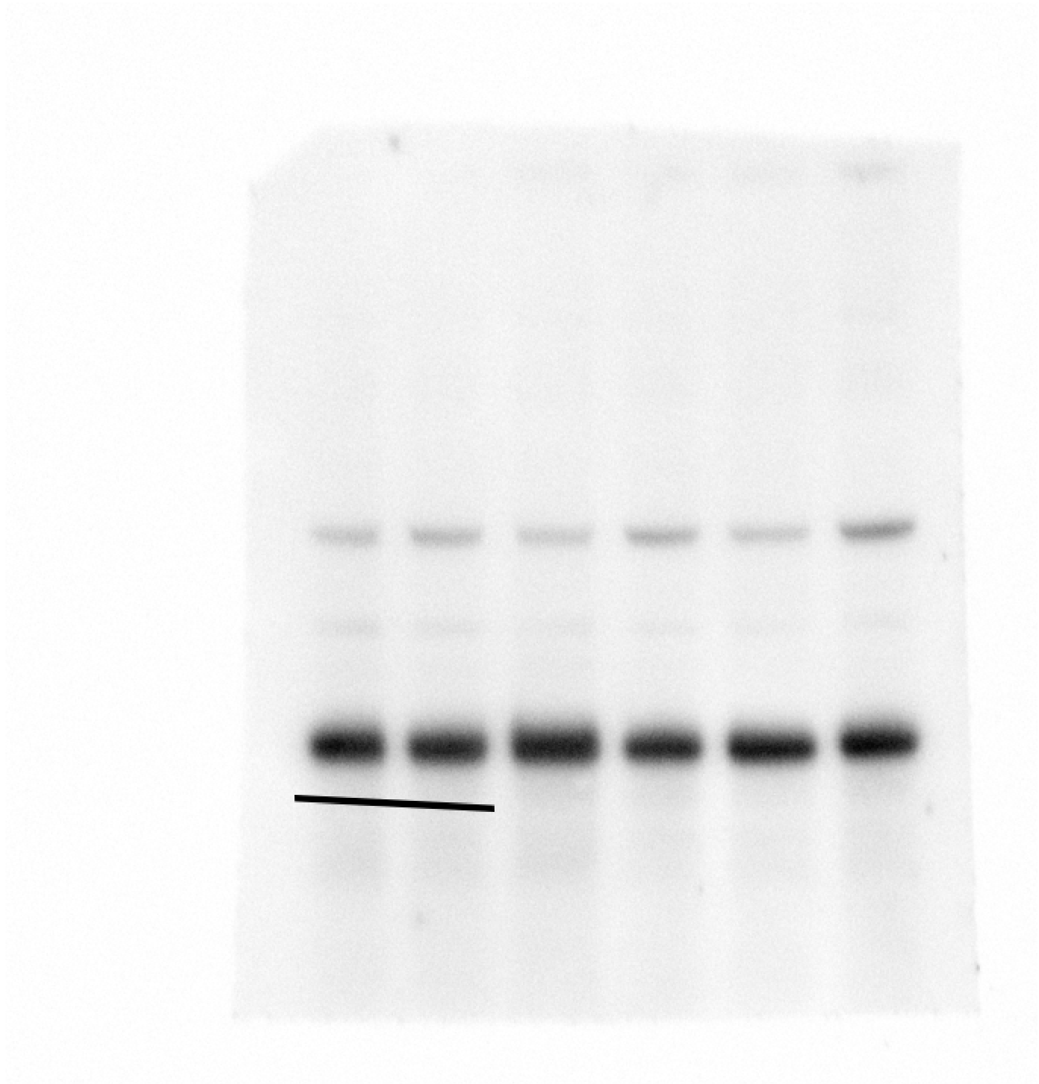

Supplement: Supplementary file 1 [file cells-13-01887-s001.zip › Supplementary File S1.pdf]
